# Evaluation of the effect of different surface treatments on the bond strength of non-precious alloy‒ceramic interface: An SEM study

**DOI:** 10.15171/joddd.2019.031

**Published:** 2019-10-07

**Authors:** Vikram M. Belkhode, Sharayu V. Nimonkar, S. R. Godbole, Pranali Nimonkar, Seema Sathe, Anjali Borle

**Affiliations:** ^1^Department of Prosthodontics and Crown & Bridge, Asst. Professor Sharad Pawar Dental College and Hospital Sawangi, Wardha; ^2^Department of Prosthodontics and Crown & Bridge, Professor Sharad Pawar Dental College and Hospital Sawangi, Wardha; ^3^Department of Oral and Maxillofacial Surgery, Asst. Professor Government Medical Hospital, Nagpur

**Keywords:** Bond failure, degassing, metal ceramic restorations, sandblasting, scanning electron microscope, surface grinding, ultrasonic cleaning

## Abstract

***Background.*** Dental porcelain has excellent esthetics in combination with biocompatibility and is one of the most commonly used restorative materials. Its low tensile strength remains a major drawback. The porcelain-fused-to-metal restorations have been introduced to increase the fracture resistance of dental porcelain. The aim of this study was to evaluate the effect of different surface treatments on the bond strength of a non-precious alloy to ceramic.

***Methods.*** The present cross-sectional observational study was conducted with forty samples of cobalt‒chromium that were fabricated with porcelain interposed between the two metal test pieces. The metal was subjected to combinations of different surface treatments. The samples group A (n=10) were not subjected to any surface treatments. Group B samples underwent sandblasting and surface grinding. Group C samples were subjected to sandblasting, surface grinding and degassing; and group D samples underwent sandblasting, surface grinding, ultrasonic cleaning and degassing. The tensile bond strength was measured in a universal testing machine, and a scanning electron microscope (SEM) was used to obtain images of the samples after surface treatment to determine the surface irregularities and after the debonding of the samples for the type of the bond failure. ANOVA was used for the statistical analysis.

***Results.*** The results showed significant variations in the tensile bond strength between the four groups (F=251.05, P=0.000). The SEM images of group A showed no surface irregularities; group C samples exhibited surface irregularities more than those in group B. Group D had the highest surface irregularities. SEM evaluations showed a statistically significant difference in the type of bond failure (P<0.001).

***Conclusion.*** Based on the results of this study, it can be concluded that the surface treatments on the metal increased the bond strength of the metal‒ceramic interface significantly. A combination of sandblasting, surface grinding and ultrasonic cleaning, followed by degassing, resulted in the highest tensile bond strength.

## Introduction

Metal‒ceramic restorations are extensively used in prosthetic dentistry as a restoration of choice. Favorable properties of porcelain, including biocompatibility, excellent abrasion resistance, color and dimensional stability, contribute to the current popularity of this esthetic veneering material. At the same time, porcelain is known for its poor tensile, shear and impact strength characteristics.^1^ To overcome these problems, the porcelain was fused with a cast alloy substructure.

Metal has a higher mechanical strength than ceramic, and hence this combination makes the restoration more fracture resistant.^2^ The recent increase in the cost of precious metals, the relatively low cost of the non-precious alloys and claims of improved physical properties and clinical success have made the non-precious metal the metal of choice for metal‒ceramic restorations.^3^ Although metal‒ceramic restorations are very popular, metal and ceramic have bonding problems. The separation of ceramic facing from the metal substructure creates an unfavorable situation for both the patients and dentists.

It has been established that surface treatment of the metal prior to the application of porcelain improves the bond strength between the porcelain and the metal.^4^ However, preparation of the ceramo-alloy surface before porcelain bonding has been a subject of controversy among dental ceramists. The literature is replete with theories regarding the effects of surface texture on the bond strength.^5^

Sandblasting is a technique of creating micro-irregularities for mechanical adhesion of the porcelain to the metal substructure by exposing the metal to aluminum oxide particles.^6^ Degassing, also commonly referred to as oxidation, outgassing and pre-oxidation, is the method used to remove the entrapped gas and to form a metal oxide layer for the chemical form of the bond between the metal and porcelain.^7^ Cleaning, using ultrasonic cleansers with a detergent or distilled water, removes the impurities over the copings, increasing the bond at metal‒porcelain interface.^8^ The acid etching treatment theoretically increases the surface area, which would be expected to enhance mechanical bonding.^9^ However, there is still no agreement regarding the best method of metal preparation for ceramic bonding. It is assumed that combining different surface treatments would enhance the bond strength at the metal‒ceramic interface.

The metal‒ceramic bond interface is critical for the functional and esthetic success of the metal‒ceramic restorations. Therefore, this in vitro study was performed to evaluate whether the different surface treatments on the metal before porcelain firing affects the bond strength of non-precious alloy‒ceramic interface. Furthermore, the types of bond failure were determined to verify the efficacy of surface treatments.

## Methods

The cross-sectional observational study was conducted for three years from May 2012 to May 2015 after the ethical clearance from the institutional ethics committee. Forty cobalt‒chromium samples with porcelain interposed between them were fabricated for the study. The sample size was determined by using a sample size formula with the desired error of margin.

### Step 1. Fabrication of the Test Pieces 

**Figure 1:**
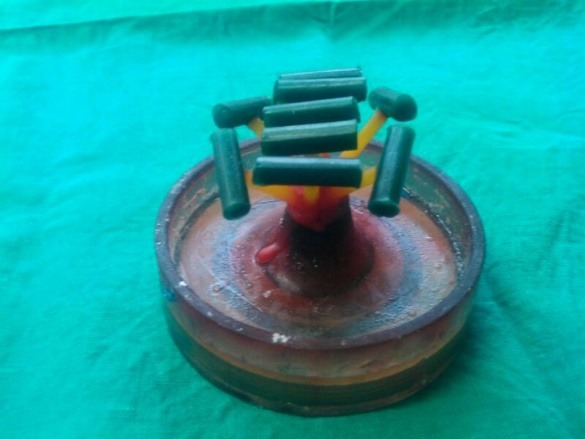
Spruing of the wax pattern.

Prefabricated sprue waxes, measuring 5 mm in diameter, were directly cut to a length of 15 mm for casting, which served as wax patterns. The use of sprue wax of the desired diameter prevented the possibility of error in the dimension. 2-mm diameter sprue wax was attached to the wax pattern (Figure 1) and invested.

Wax elimination, casting, divesting of the casting, finishing and polishing of the metal test pieces (Co-Cr) (Bego) were carried out in a conventional manner.Eighty test pieces were fabricatedto form the forty samples.

### Step 2. Grouping of the Samples 

The test pieces were randomly selected and the end surface of the test pieces, where the porcelain was to be applied, was subjected to different combinations of the surface treatments. The samples were divided into four groups (n=10). The samples of in group A were not subjected to any surface treatment (control group). The test pieces in group B were subjected to sandblasting and surface grinding; the test pieces in group C were subjected to sandblasting, surface grinding and degassing; and the test pieces in group D underwent sandblasting, surface grinding, ultrasonic cleaning and degassing.

### Step 2. Surface Treatments 

### Sandblasting 

The samples were sandblasted on the end surfaces of the test piece with 50-µm aluminum oxide particles in a sandblasting unit (Duosand UCIN, Dentaire).

### Surface Grinding 

The test pieces were subjected to surface grinding on the end surface where the porcelain was applied. A carbide bur with a diameter of 0.5 mm (DFS) was selected, and a unidirectional grinding was carried out.

### Degassing

The samples were placed in a ceramic furnace (Dentsply) and heated to 1950°F and held at this temperature in a 28-inch (64‒70 cm) mercury vacuum for 5 minutes. It formed the oxide layer that helped in the chemical bonding of the metal to ceramic. Degassing was carried out at last so as not to disturb the oxide layer.

### Ultrasonic Cleansing 

The test pieces were cleaned in an ultrasonic cleaner (CD-4820) with distilled water for 10 minutes.

### Step 3. Fabrication of Samples

Once all the test pieces were treated with the desired combinations of the surface treatments, the test pieces were subjected to the porcelain firing to form samples. The following steps were followed.

### Customizing the Sagger Trays (Figure 2)

**Figure 2:**
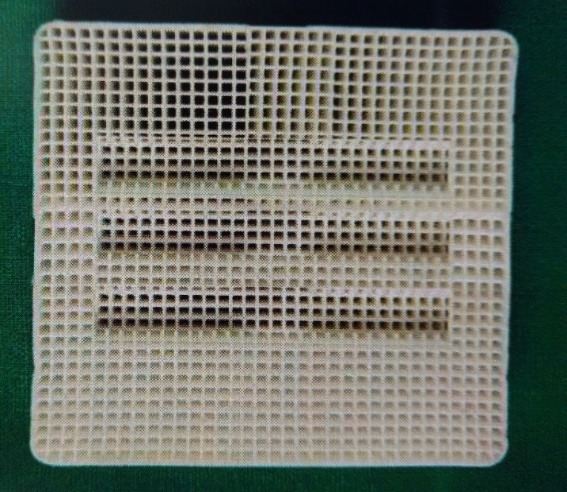
The customized sagger tray.

The sagger tray (firing tray) was customized to support and stabilize the test pieces. The tray was grooved along the length of the test piece to stabilize the test pieces. Another groove was placed on the end surfaces of the test piece so that the two test pieces could be placed parallel with a gap of 2 mm between the ends for the porcelain application. This helped in the standardization of the thickness of the porcelain and also the paralleling of the samples as any bend would create a fulcrum, leading to the fracture of the samples under a slight tensile load.

### Porcelain Application 

**Figure 3:**
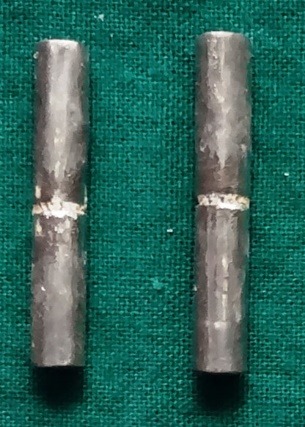
The fabricated samples.

The test pieces were placed on customized sagger trays facing each other, with the surfaces having undergone surface treatments; during the placement, a 2-mm gap was maintained for porcelain firing. The samples were air-dried in front of the open furnace chamber for 10 minutes; then, the opaque layer of the porcelain was applied and heated from 1250ºF to 1750ºF at a rate of 80ºF/minute. Two test pieces formed a sample with a 2-mm-thick porcelain piece interposed between them (Figure 3).

### Step 4. Testing of the Samples

### Testing the Samples with a Universal Testing Machine 

**Figure 4:**
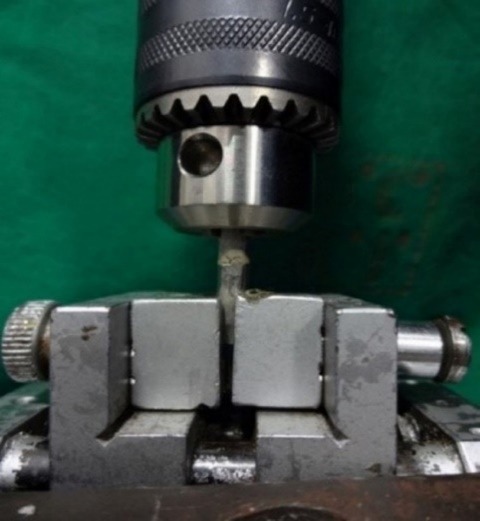
Holding of the sample in the fixture.

The samples of the metal block, with ceramic interposed, were placed in the fixtures of the universal testing machine (Instron, India Private Limited) and a maximum tensile load was applied at a crosshead speed of 5 mm/min (Figure 4) and the tensile bond strength for all the four groups was tabulated (Table 1).

**Table 1:** Comparison of tensile bond strength (MPa) of groups A, B, C and D

**Group**	**N**	**Mean**	**SD**	**Std. error**	**95% Confidence interval for mean**		**Min**	**Max**
					**Lower bound**	**Upper bound**		
A	10	14.51	1.62	0.51	13.35	15.66	12.00	16.80
B	10	22.80	2.55	0.80	20.97	24.62	18.00	26.00
C	10	32.73	1.93	0.61	31.342	34.11	30.00	36.50
D	10	44.22	1.86	0.58	42.88	45.55	41.10	47.00

### Testing the Samples under a Scanning Electron Microscope (SEM) 

A scanning electron microscope (Jeol JSM-6380A) was used for obtaining images of the samples by scanning it with a high-energy beam of electrons. Since ceramic is non-conductive, it should be coated with an ultrathin coating of electrically-conductive gold material in a sputtering device.

The samples in each group were scanned after surface treatment to determine the surface roughness and after debonding to determine the type of bond failure.

ANOVA and chi-squared test were used for the statistical analysis of the results (Tables 2 and 3), using SPSS 22.0 and P<0.05 was considered as level of significance.

**Table 2:** One-way ANOVA

**Source of variation**	**Sum of squares**	**Df**	**Mean square**	**F**	**P-value**
**Between groups**	2298.13	2	1149.06	251.05	0.000
**Within groups**	123.57	27	4.57		
**Total**	2421.71	29			

**Table 3:** Type of bond failure in the four study groups (chi-squared test)

**Group**	**Adhesive**	**Cohesive**	**Mixed** **(Adhesive + Cohesive)**	**Total**
**Group A**	8 (80%)	-	2 (20%)	10
**Group B**	-	7 (70%)	3 (30%)	10
**Group C**	-	9 (90%)	1 (10%)	10
**Group D**	-	10 (100%)	-	10
**Total**	8 (20%)	26 (65%)	6 (15%)	40
**2א-value**	5.90
**P-value**	0.20, NS, P>0.05			

## Results

The results showed significant differences in the tensile bond strength between the four groups (F=251.05, P=0.000).

The mean tensile bond strength in group A was 14.51±1.62. Of 10 samples of group A, 80% showed adhesive, and 20% had a mixed type of bond failure. Chi-squared test showed significant differences in the type of bond failure in group A (P<0.0001).

The mean tensile bond strength in group B was 22.80±2.55. Of 10 samples in group B, 30% exhibited mixed, and 70% had a cohesive type of bond failure. Chi-squared test revealed significant differences in the type of bond failure in group B (P<0.0001).

The mean tensile bond strength fin group C was 32.73±1.93. Of 10 samples in group C, 10% had mixed, and 90% had a cohesive type of bond failure. Chi-squared test showed significant differences in the type of bond failure in group C (P<0.0001).

The mean tensile bond strength in group D was 44.22±1.86. Of 10 samples in group D, 0% exhibited adhesive and mixed, and 100% had cohesive bond failure. Chi-squared test revealed significant differences in the type of bond failure in group D (P<0.0001).

SEM showed a statistically significant difference in the type of bond failure.

**Figure 5:**
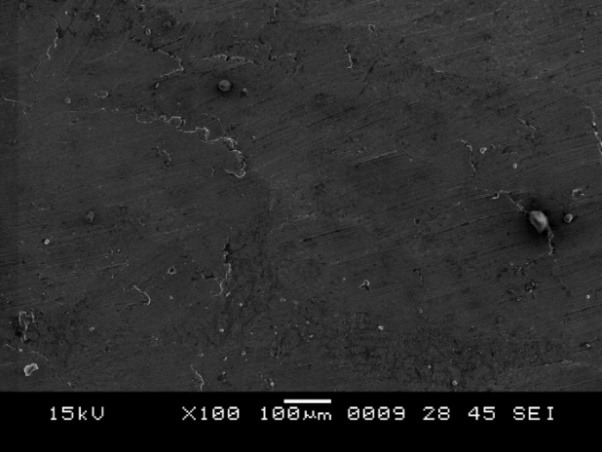
SEM image showing surface irregularities after surface treatment in group A.

**Figure 6:**
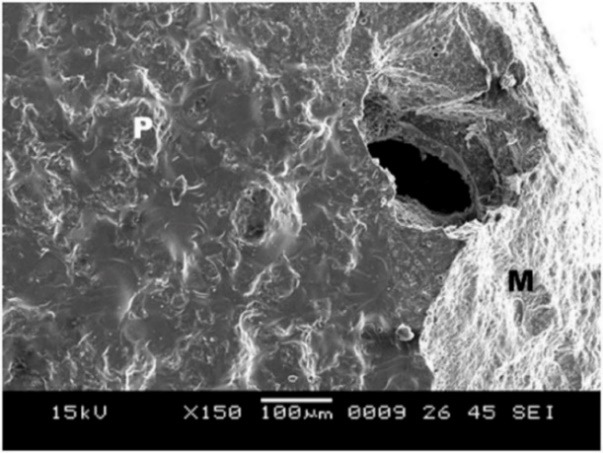
SEM image of the debonded surface in group A, where P is porcelain and M is metal.

**Figure 7:**
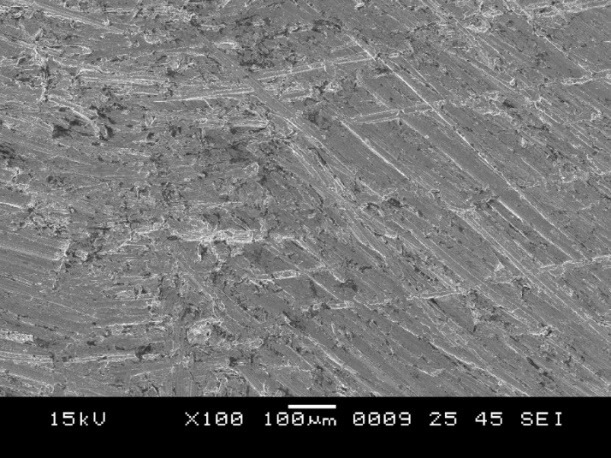
SEM image showing surface irregularities after surface treatment in group B.

**Figure 8:**
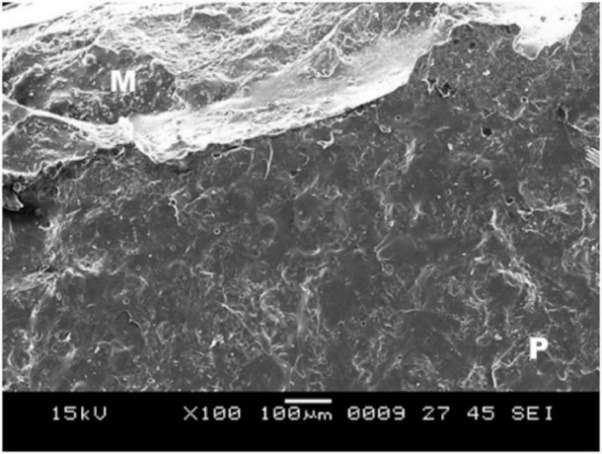
SEM image of the debonded surface in group B, where P is porcelain and M is metal.

**Figure 9:**
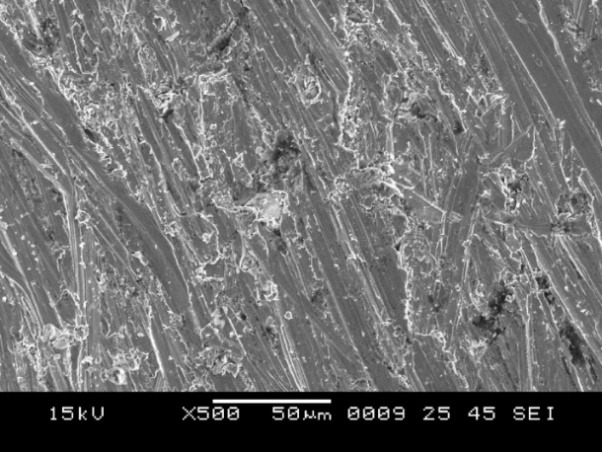
SEM image showing surface irregularities after surface treatment in group C.

**Figure 10:**
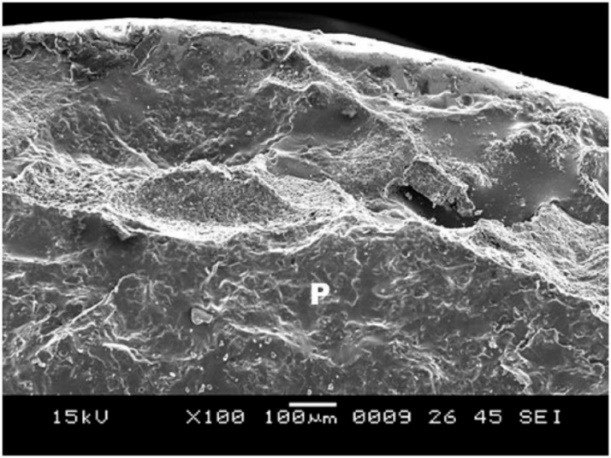
SEM image of the debonded surface in group C, where P is porcelain and M is metal.

Within the scope and limitations of this study, the following observations were made:

1. The surface treatments definitely increased the bond strength of a non-precious alloy ceramic interface.

2. The tensile bond strength was the highest in group D and the lowest in group A. Group B and C showed higher bond strength compared to Group A, with lower bond strength compared to group D.

3. Scanning electron microscope observations in group A after surface treatment showed a smooth surface with no irregularities (Figure 5), and the type of bond failure was predominately adhesive (Figure 6).

4. Group B showed surface irregularities, which were less than that in group C (Figure 7), with a mixed type of bond failure that was a combination of adhesive and cohesive failures, predominately adhesive (Figure 8).

5. Group C showed surface irregularities, which were more than those in group B (Figure 9). The type of bond failure was cohesive, but the line of demarcation was not clean (Figure 10).

**Figure 11:**
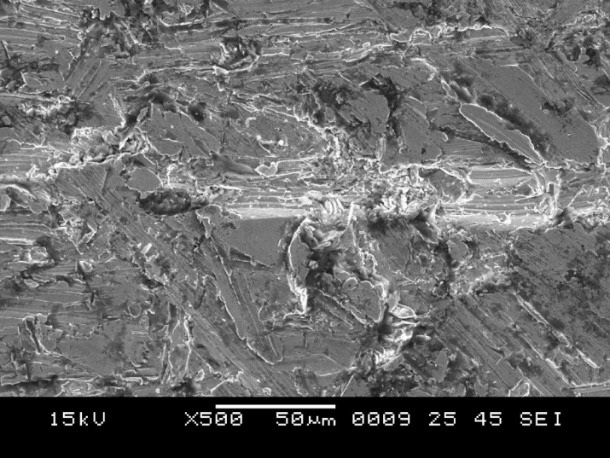
SEM image showing surface irregularities after surface treatment in group D.

**Figure 12:**
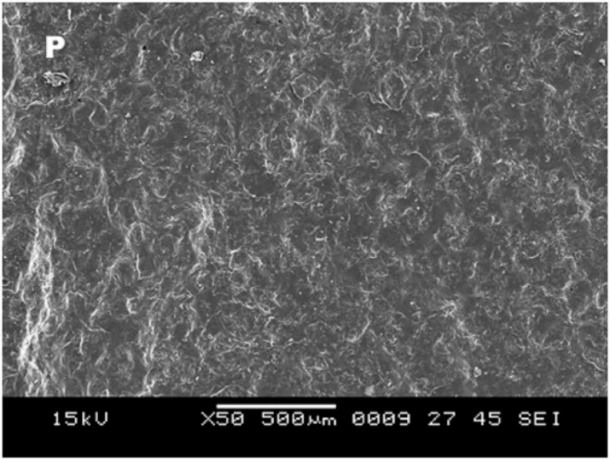
SEM image of the debonded surface in group D, where P is porcelain and M is metal.

Group D showed surface irregularities, which were more than those in all the other groups (Figure 11); this is essential for bonding between the metal and ceramic and cohesive type of bond failure with a clean line of demarcation (Figure 12).

## Discussion

Metal‒ceramic restorations have extensively been used in restorative dentistry for many years. Metal‒ceramic restorations combine the beauty of porcelain and the strength of a metal substructure. Bonding of porcelain to metal supports the porcelain with a metal substructure and increases the strength of the porcelain. Many non-precious alloys are being specifically designed for metal‒ceramic restorations. Non-precious metals have high strength, are less bulky, exhibit less thermal transmission and have fusion properties that make these alloys the preferred metal for porcelain substructures.^10^

Ronald et al^11^ studied the bond strength of porcelain with precious, semiprecious and non-precious alloys and reported that the bond strength was highest for non-precious alloys. Cobalt‒chromium was used in this study as it is also gaining popularity as it is a nickle- and beryllium-free alloy.

The success of metal‒ceramic restorations depends on the metal‒ceramic bond which is dictated by the chemical bond (oxide layer formation), mechanical interlocking, van der Waals forces and compressive forces originating from the coefficient of thermal expansion.

Wagner et al^12^ reported that the greater the roughness of the metal surface, the higher the bond strengths. The surface roughness of the metal can be achieved by sandblasting, surface grinding and acid etching. Sandblasting creates micro-irregularities in the casting that increase mechanical retention. Carpenter and Goodkind,^13^ reported the advantages of sandblasting, reiterating that it enhances the wettability of the metal by porcelain.

Surface grinding results in the micro-locking between the porcelain and metal, increasing mechanical retention. Van Noort^14^ recommended that multi-directional grinding can entangle debris and air in the surface irregularities. This entangled debris and air are further decomposed on firing and form gas bubbles at the metal‒ceramic interface, leading to reduced bond strength. Therefore, a unidirectional grinding was opted to achieve a debris-free metal. Grinding was carried out, in this study, before degassing so as not to disturb the oxide layer.

The only and the most significant mechanism of porcelain‒metal attachment is a chemical bond between the porcelain and the oxide on the surface of the metal substructure. Graham et al^15^ reported that any surface treatment after degassing disturbs the oxide layer; therefore, in this study degassing was carried out after surface grinding, sandblasting and ultrasonic cleansing, which resulted in the highest bond strength.

Lahori et al^16^ published a study in 2014, in which they compared the effect of seven different alloy surface treatments on the bond strength of the porcelain‒metal interface. They found the bond strength to be the lowest for the group that was steam-cleaned. The possible reason for this was the entrapped air bubbles and the contaminants in the ultrasonic cleanser that got trapped in the irregularities. Therefore, the ultrasonic cleaner was periodically checked to ensure pure steam.

Naylor^17^ also reported that one of the reasons for the porcelain‒metal bond failure is contamination, and the best procedure to remove the impurities is ultrasonic cleaning in distilled water for 10 minutes. The absence of a clear line of demarcation in group C is attributed to the impurities present, which were not found in group D.

The SEM images also supported the statement that surface roughness increases the bond strength; the porcelain remained on the rough-textured surface of the metal that shows a cohesive type of the bond failure compared to the smooth surface-textured metal that shows adhesive bond failure.

There exists a need for further research to elucidate the bond strength and type of bond failure with larger sample size and more advanced equipment, which proved to be the limitations of this study. There is scope for further studies to evaluate the surface roughness, using more sophisticated tools, including profilometry, 3D scanning microscopy or confocal laser scanning microscopy (CLSM) for better analysis. The success of metal‒ceramic restorations depends on the firmness of the ceramic bond over the metal. In this study, an attempt was made to evaluate the effect of different surface treatments on the bond strength of the metal ceramic interface for the long-term functional and esthetic success of metal‒ceramic restorations.

## Conclusion

Based on the results of this study, it can be concluded that the surface treatments on the metal increase the bond strength of the metal‒ceramic interface. The finding was supported by SEM images that showed a highly rough metal surface after the surface treatment, which is essential for the mechanical interlocking between the metal and porcelain to increase the bond strength.

## Competing Interests

The authors declare no conflict(s) of interest related to the publication of this work.

## Authors’ Contributions

VMB and SVN contributed to the concept and design of the study and drafting of the manuscript. SRG and PN interpreted the data for the work and drafted the manuscript critically. The rest of the authors were involved in revising the manuscript for important intellectual content.

## Acknowledgments

Authors wishes to acknowledge the help, support and permission of mechanical department of VNIT Nagpur and the teaching, non-teaching staff of the Department of Prosthodontics SPDC.

## Funding

Not applicable.

## Ethics Approval

The study protocol was approved by the Institutional Ethics Committee of Sharad Pawar Dental college and hospital (Ref.No. DMIMS (DU) / IEC/2012-13/844).

## References

[1] Emilija Bajraktarova-Valjakova, Vesna Korunoska-Stevkovska, Biljana Kapusevska, Nikola Gigovski1, Cvetanka Bajraktarova-Misevska, Anita Grozdanov (2018). Contemporary Dental Ceramic Materials, A Review: Chemical Composition, Physical and Mechanical Properties, Indications for Use. Open Access Maced J Med Sci.

[2] Juliano Milczewsky Scolaro, Jefferson Ricardo Pereira, Accacio Lins do Valle, Gerson Bonfante, Luiz Fernando Pegoraro (2007). A Comparative study of ceramic-to-metal bonding. Braz Dent J.

[3] Kelly J, Robert Robert, Rose C Thomas (1983). Non precious alloys for use in Fixed Prosthodontics- A literature review. J Prosthet Dent.

[4] Vaidya A Nitin, Parkhedkar RD (2010). The Effect of Three Methods of Surface Treatments on Flexural Bond Strength of Nonprecious Alloy-ceramic Interface; Evaluated by Four Point Bend Test and Scanning Electron Microscopic Analysis. JIDA.

[5] Belwalkar Belwalkar, VR VR, Gade Gade, J J, Mankar Mankar, NP NP (2016). Comparison of the effect of shear bond strength with silane and other three chemical presurface treatments of a glass fiber-reinforced post on adhesion with a resin-based luting agent: An in vitro study. Contemp Clin Dent.

[6] Mehulic Ketij, Laus-Sosic Martina, Schauperl Zdravko, Vojvodic Denis, Stefancic Sanja (2009). Influence of cast surface finishing process on metal-ceramic bond strength. J Prosthet Dent.

[7] Rathi Shraddha, Parkash Hari, Chttaranjan B, Bhargava Akshaya (2011). Oxidation heat treatment affecting metal ceramic bond. Indian J Dent Res.

[8] Musani S, Musani I, Dugal R, Habbu N, Madanshetty P, Virani D (2013). An in vitro Comparative Evaluation of Micro Tensile Bond Strength of Two metal bonding Resin Cements bonded to Cobalt Chromium alloy. J Int Oral Health.

[9] Park WU, Park HG, Hwang KH, Zhao J, Lee JK (2017). Interfacial Property of Dental Cobalt–Chromium Alloys and Their Bonding Strength with Porcelains. J Nanosci Nanotechnol.

[10] Jadhav VD, Motwani BK, Shinde J, Adhapure P (2017). Comparative evaluation of conventional and accelerated castings on marginal fit and surface roughness. Contemp Clin Dent.

[11] Ronald P (1977). Lubovich and Richard J Goodkind Bond strength studies of precious, semiprecious and non-precious ceramic-metal alloy with two porcelain. J Prosthet Dent.

[12] Wagner WC, Asgar K, Bigelow WC, Flinn RA (1993). Effect of interfacial variables on metal porcelain bonding. J Biomed Mater Res.

[13] Carpenter A (1979). Carpenter AMichael, Goodkind JRichardEffect of varying surface texture on bond strength of one semiprecious and one nonprecious ceramo-alloy. J Prosthet Dent.

[14] Noort R. Introduction to Dental Materials. London: Mosby; 1994. p. 215-217.

[15] Graham JD, Johnson A, Wildgoose DG, Shareef MY, Cannavina G (1999). The Effect of Surface Treatments on the Bond Strength of a Nonprecious Alloy- Ceramic Interface methods. Int J Prosthodont.

[16] Lahori Manesh, Nagrath Rahul, Sisodia Siddharth Sisodia Siddharth, Dagar Preety Dagar Preety (2014). The Effect of Surface Treatments on the Bond Strength of a Nonprecious Alloy–Ceramic Interface: An Invitro Study. J Indian Prosthodont Soc.

[17] Naylor PW. Introduction to Metal Ceramic Technique. Quintessence publishing Co Inc: U.S; 1992. p. 83-113.

